# Treatment of Mixed Dry Eye Disease with Topical Azithromycin: A Prospective Controlled Pilot Study

**DOI:** 10.3390/jcm15197549

**Published:** 2026-09-28

**Authors:** Blaž Cestnik, Ana Dovžak Fritz, Tomaž Bone, Spela Stunf Pukl

**Affiliations:** 1 Faculty of Medicine, University of Ljubljana, Vrazov trg 2, 1000 Ljubljana, Slovenia; 2 Eye Hospital, University Medical Centre Ljubljana, Grablovičeva 46, 1000 Ljubljana, Slovenia

**Keywords:** dry eye disease, mixed dry eye disease, azithromycin, meibomian gland dysfunction, ocular surface, tear film, inflammation

## Abstract

**Background:** This study evaluated the efficacy of topical azithromycin ophthalmic solution (Azyter^®^, 15 mg/g) as second-line treatment for mixed dry eye disease (DED), given the drug’s known non-antimicrobial anti-inflammatory and immunomodulatory properties. **Methods:** In this pilot, prospective, controlled, non-randomized interventional study, fifteen patients with mixed DED refractory to first-line treatment received topical azithromycin for 30 days (twice daily for 3 days, then once daily for 27 days); ten healthy individuals served as controls. Clinical evaluation was performed before and after treatment in the study group and once in controls, and included the Dry Eye Questionnaire-5 (DEQ-5), Schirmer test, non-invasive tear break-up time (NITBUT, Sirius PLUS corneal topography), tear meniscus height (TMH; Sirius PLUS corneal topography), Efron scale grading of blepharitis, meibomian gland dysfunction (MGD), and conjunctival hyperemia; Oxford-scale grading of corneal fluorescein staining, and corneal epithelial thickness (swept-source anterior segment optical coherence tomography, AS-SS-OCT). **Results:** After treatment, DEQ-5 scores improved significantly (*p* < 0.001) and no longer differed from controls. Schirmer test and NITBUT values increased significantly (*p* = 0.003 and *p* = 0.027, respectively), becoming comparable to controls. Blepharitis, MGD, and corneal fluorescein staining scores also improved significantly (*p* < 0.001, *p* < 0.001, *p* = 0.002), as did maximum corneal epithelial thickness (*p* = 0.037). **Conclusions:** In this pilot cohort, second-line treatment of mixed DED with topical azithromycin significantly improved disease symptoms and signs, supporting its potential as a treatment option. Larger randomized studies are needed to confirm these findings and determine the optimal treatment regimen and duration.

## 1. Introduction

Dry eye disease (DED) is a multifactorial disorder of the ocular surface characterized by a loss of tear film and/or ocular surface homeostasis. Its pathophysiology involves tear film instability, hyperosmolarity, ocular surface inflammation, and neurosensory abnormalities [1].

Epidemiological estimates vary, the prevalence is up to 12.8% in White populations and 23.7% in East Asian populations, and it is higher in women and increases with age [2,3,4].

Patients with DED experience eye dryness, foreign body sensation, burning, itching, or irritation, as well as blurred and fluctuating vision, which can impair the quality of life, reduce work productivity, and impose a considerable socioeconomic burden [5,6].

DED has been classified into aqueous-deficient and evaporative subtypes. However, these mechanisms most frequently coexist, resulting in mixed DED. Evaporative DED is most commonly associated with meibomian gland dysfunction (MGD), which disrupts the lipid layer of the tear film, leading to increased tear evaporation and tear film instability, whereas aqueous-deficient DED results from impaired lacrimal gland secretion [7,8,9,10].

Regardless of the initiating mechanism, the underlying pathogenic processes converge on tear film instability, hyperosmolarity, subsequent cellular stress in the ocular surface epithelium, triggering inflammatory pathways and perpetuating the disease process [11].

In addition, several structural changes of the corneal epithelium can be associated with DED. Previous studies have assessed corneal epithelial thickness in DED using swept-source anterior segment optical coherence tomography (AS-SS-OCT). However, the findings are inconsistent, and the role of corneal epithelial thickness as a biomarker of disease severity and treatment response remains unclear [12,13,14,15,16].

The primary goal of DED management is to restore tear film and ocular surface homeostasis, thereby interrupting the vicious cycle of tear film instability, hyperosmolarity, inflammation, and epithelial damage [17]. First-line management includes patient education, lifestyle and environmental modifications, tear supplementation with artificial tears, and eyelid hygiene [17]. Thereafter, for patients with predominant inflammatory or immune-mediated disease, topical anti-inflammatory therapies, including corticosteroids and cyclosporine A, are recommended [17,18,19,20]. Despite the availability of therapeutic options, treatment of DED remains challenging in some patients because adequate and sustained improvement in symptoms and clinical signs is not always achieved. Consequently, alternative therapies and approaches, especially those targeting the underlying inflammation, are an important focus of current research [17].

Azithromycin is a macrolide antibiotic that, in addition to its antimicrobial activity, exhibits important anti-inflammatory and immunomodulatory properties [21]. It modulates multiple components of the inflammatory response by suppressing the production of pro-inflammatory cytokines, including interleukin (IL)-1β, IL-6, IL-8, and tumor necrosis factor-α (TNF-α), reducing neutrophilic inflammation, and inhibiting the activation of the nuclear factor kappa B (NF-κB) signaling pathway [22,23,24,25]. Several clinical studies have also demonstrated that topical azithromycin is effective in the treatment of anterior and posterior blepharitis, as well as blepharitis-associated DED, improving both clinical signs and symptoms while exhibiting a favorable safety profile with only mild, transient ocular adverse effects [26,27,28,29,30,31,32,33,34].

Given its anti-inflammatory and immunomodulatory properties and favorable safety profile in the treatment of MGD, topical azithromycin may represent a promising therapeutic option for patients with mixed DED.

However, to the best of our knowledge, no prospective study has specifically evaluated the efficacy of topical azithromycin in patients with mixed DED or changes in corneal epithelial thickness following treatment. Therefore, the aim of the present prospective controlled pilot study was to evaluate the efficacy of topical azithromycin in patients with mixed DED by assessing changes in subjective symptoms and objective ocular surface parameters, including tear production, tear film stability, corneal fluorescein staining, blepharitis, MGD, and corneal epithelial thickness, before and after treatment.

## 2. Materials and Methods

### 2.1. Study Design and Setting

This was a prospective, non-randomized, controlled interventional pilot study conducted at the Eye Hospital, University Medical Centre Ljubljana, Slovenia, from October 2025 to July 2026. The efficacy of topical azithromycin was evaluated by comparing subjective symptoms and objective ocular surface parameters before and after treatment. Treatment effects were evaluated by comparing baseline and post-treatment measurements within the treatment group. Baseline measurements were additionally compared with those of a healthy control group. The study protocol was approved by the National Medical Ethics Committee of the Republic of Slovenia (Approval No. 0120-299/2025-2711-3). All participants provided written informed consent prior to study enrollment, and the study was conducted in accordance with the principles of the Declaration of Helsinki.

### 2.2. Study Population

The study included 15 patients with clinically confirmed mixed DED and already managed with the first-line treatment of DED, who were treated with topical azithromycin and ten healthy participants without signs or symptoms of DED who served as a control group. The demographic characteristics of the study participants are presented in Table 1. Eligible patients were adults (≥18 years) with clinically confirmed mixed DED, defined by a Schirmer test result <10 mm/5 min and a non-invasive tear break-up time (NITBUT) < 10 s, an inadequate response to first-line treatment, and written informed consent. Exclusion criteria were: (1) ocular trauma; (2) active ocular infection; (3) non-inflammatory ocular diseases; (4) ocular inflammation unrelated to DED; (5) ocular surgery within the previous six months; (6) previous treatment with topical or systemic medications for MGD or DED, including topical or oral antibiotics, topical cyclosporine A, topical or oral corticosteroids, topical non-steroidal anti-inflammatory drugs, and topical anti-allergic medications; (7) pregnancy; (8) uncontrolled systemic disease; (9) conditions that could interfere with adherence to the study protocol; (10) allergy to study medication; (11) inability to provide written informed consent or comply with the study protocol.

As this was designed as a pilot study, the target sample size was prospectively set at 15 patients. No formal a priori power calculation was performed. The sample size was determined based on the feasibility of recruiting eligible patients with mixed DED within the planned study period. The sample was intended to provide preliminary estimates of treatment effects and outcome variability and to inform the design of future adequately powered studies.

### 2.3. Treatment Protocol

Patients received topical azithromycin (Azyter^®^ 15 mg/g, Laboratoires Théa, Clermont-Ferrand, France) twice daily for the first 3 days, followed by once-daily administration for the remainder of the one-month treatment period. Healthy control participants did not receive any treatment and underwent a single baseline examination. All patients were instructed to continue standard eyelid hygiene, consisting of warm compresses, gentle lid massage directed toward the lid margin, and lid cleansing with commercially available eyelid cleansing products. In addition, all patients used preservative-free artificial tears throughout the study period. Clinical examinations were performed at baseline and after one month of treatment.

### 2.4. Clinical Assessment

All participants had a baseline standard eye exam and additional tests assessing the ocular surface. Namely, the symptoms of DED were assessed using the Dry Eye Questionnaire-5 (DEQ-5) translated to native language; the same professionally translated but not fully validated version of DEQ-5 was used for all participants [35]. Validations of the translated variant followed the present study to be used for further research and clinical work. Tear production was measured by the Schirmer test I—i.e., without topical anesthetic. Additionally, diagnostic procedures oriented to the ocular surface were undertaken. The tear meniscus height (TMH) and non-invasive tear break-up time (NITBUT) were measured with the Sirius+ corneal topographer (CSO, Florence, Italy). The signs of i. blepharitis, ii. meibomian gland dysfunction (MGD), iii. the stage of conjunctival hyperemia, and iv. the grade of corneal and/or conjunctival fluorescein staining were assessed on Sirius+ high-resolution images and using Efron (i.–iii.) and Oxford (iv.) grading schemes [36,37]. To measure the corneal epithelial thickness a swept-source anterior segment optical coherence tomography (AS-SS-OCT) corneal image was obtained (Anterion, Heidelberg Engineering GmbH, Heidelberg, Germany) with the Cornea app. The mean epithelial thickness, minimum and maximum epithelial thickness, superior–inferior epithelial thickness difference, and corneal epithelial thickness variability index were measured.

After the treatment, the exam and tests were repeated in the test group, whereas the healthy control participants underwent a single baseline examination.

### 2.5. Outcome Measures

The primary outcomes in the treatment group were the improvement in the DEQ-5 score, the improvement of the Schirmer test, and the improvement of the NITBUT after one month of treatment as compared to the baseline values and compared to the healthy control group.

Secondary outcomes in the treatment group included augmentation of the TMH, improvement of signs of blepharitis, improvement of status of MGD, diminished conjunctival hyperemia, less corneal and/or conjunctival fluorescein staining, as well as any changes in the AS-SS-OCT measured corneal epithelial thickness parameters.

### 2.6. Statistical Analysis

Statistical analysis was performed using IBM SPSS Statistics version 32.0.0.0 (IBM Corp., Armonk, NY, USA). Normally distributed continuous variables were summarized as mean ± standard deviation (SD), whereas non-normally distributed continuous and ordinal variables were summarized as median with interquartile range (IQR; Q1–Q3). For the paired *t*-test, the mean difference with its 95% confidence interval (CI) was also reported. The normality of paired differences was assessed using the Shapiro–Wilk test and Q–Q plots. Normally distributed differences were analyzed using the paired Student’s *t*-test, whereas non-normally distributed differences and ordinal outcomes were analyzed using the Wilcoxon signed-rank test.

For comparisons between patients after treatment and the healthy control group, normality of the distributions in both groups and homogeneity of variances were assessed using Levene’s test. Normally distributed variables were analyzed using the independent-samples *t*-test, with Welch’s *t*-test used when variances were unequal. Non-normally distributed and ordinal variables were analyzed using the Mann–Whitney U test. Age was compared using the Mann–Whitney U test.

Hedges’ g with 95% CIs was calculated for DEQ-5, Schirmer test, NITBUT, and the four corneal epithelial thickness parameters. Missing data were not imputed. All tests were two-sided, with a *p* value < 0.05 considered statistically significant.

## 3. Results

### 3.1. Participant Characteristics

A total of 15 patients with mixed DED and 10 healthy control participants were included in the study. The treatment group had a mean age of 65.7 ± 13.9 years, whereas the healthy control group had a mean age of 57.7 ± 15.6 years. Six patients (40%) and four healthy controls (80%) were male. Participant characteristics are summarized in Table 1.

### 3.2. Primary Outcomes

**Figure 1:**
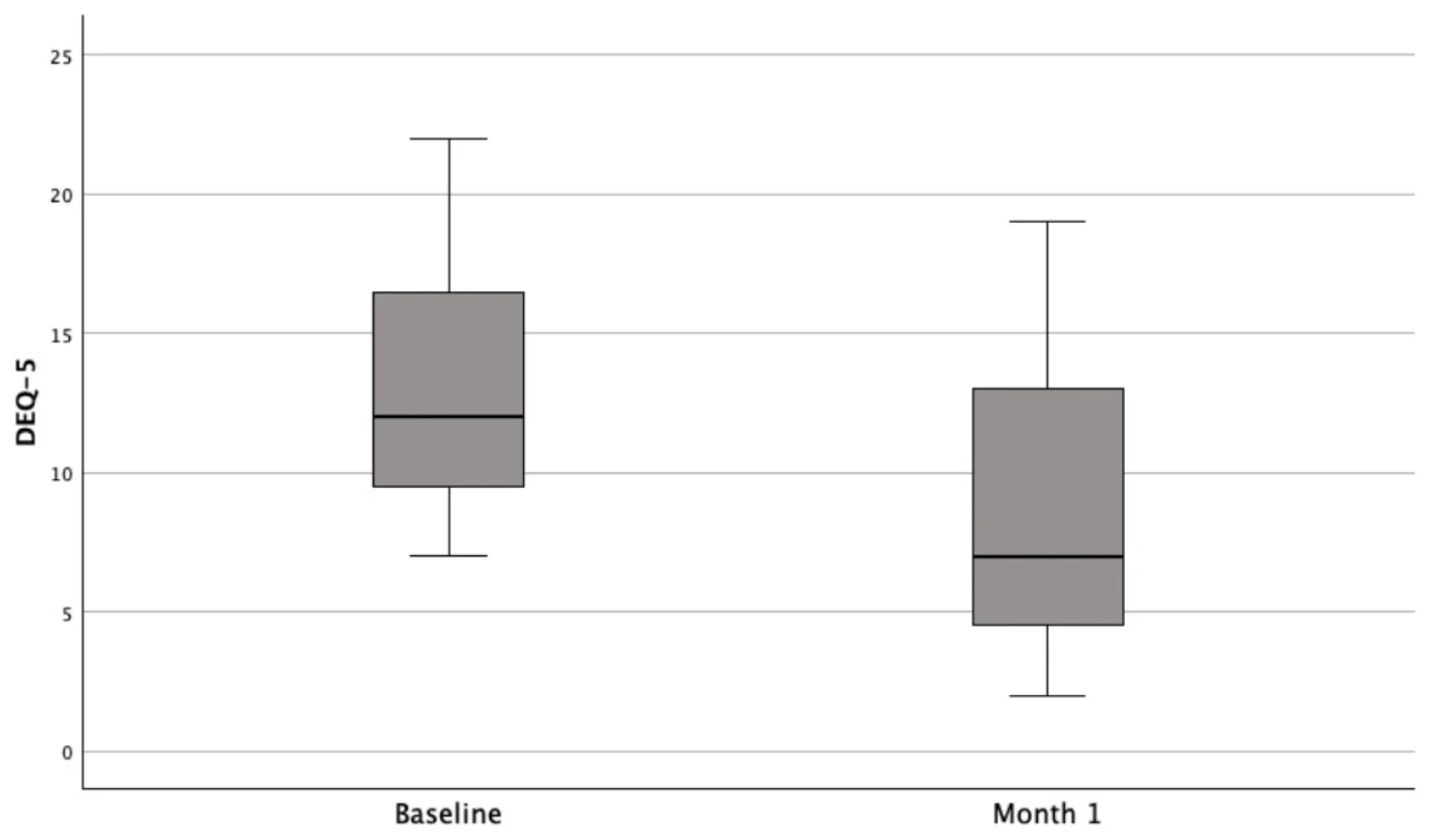
Boxplots of Dry Eye Questionnaire-5 (DEQ-5) scores at baseline and after one month of topical azithromycin treatment.

**Figure 2:**
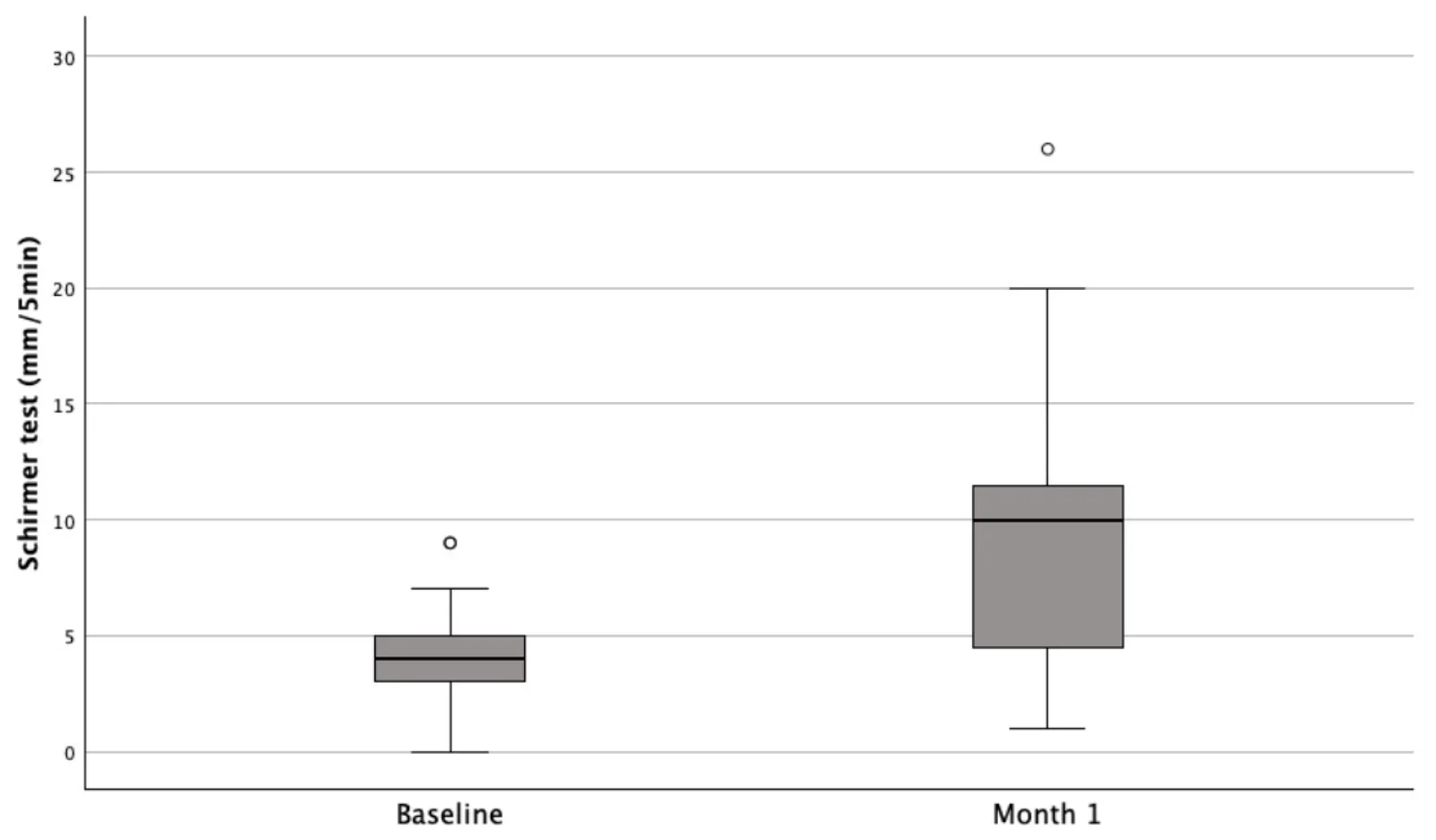
Boxplots of Schirmer test values at baseline and after one month of topical azithromycin treatment.

**Figure 3:**
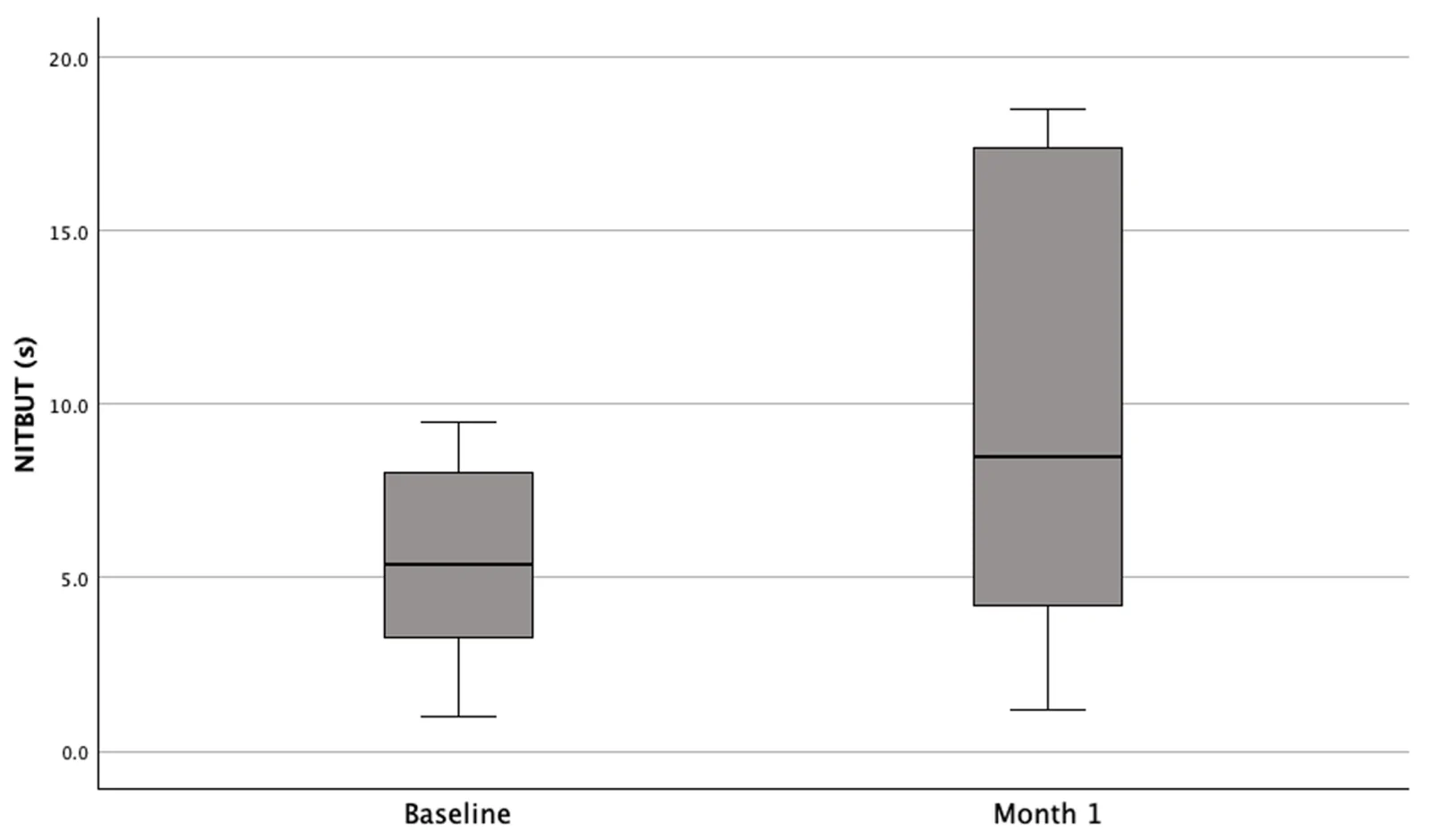
Boxplots of non-invasive tear break-up time (NITBUT) at baseline and after one month of topical azithromycin treatment.

After one month of topical azithromycin treatment, DEQ-5 scores decreased significantly from 13.00 ± 5.07 at baseline to 8.67 ± 5.21 after treatment (*p* < 0.001). Schirmer I test results increased from 4.27 ± 2.60 mm to 9.93 ± 6.84 mm (*p* = 0.003), while NITBUT increased from 5.49 ± 2.81 s to 10.01 ± 6.48 s (*p* = 0.027) (Table 2; Figure 1, Figure 2 and Figure 3). The reduction in DEQ-5 scores showed a large standardized effect size, while the improvements in the Schirmer I test and NITBUT showed large and moderate standardized effect sizes, respectively (Table 3).

### 3.3. Secondary Outcomes

#### 3.3.1. Clinical Signs

Significant improvements were observed in signs of blepharitis and Meibomian gland dysfunction as assessed by the decreased grades on Efron scale (blepharitis, *p* < 0.001, MGD *p* < 0.001), as well as in decreased corneal fluorescein staining assessed by the Oxford scale (*p* = 0.002), after one month of treatment. Conjunctival hyperemia showed a non-significant trend toward improvement (*p* = 0.063) (Table 4).

#### 3.3.2. Tear Meniscus Height

No significant change in the TMH was observed after one month of treatment (0.36 ± 0.18 mm at baseline vs. 0.36 ± 0.17 mm after treatment; *p* = 0.960) (Table 5).

#### 3.3.3. Corneal Epithelial Thickness

The maximum corneal epithelial thickness increased from 59.10 ± 5.67 µm at baseline to 64.30 ± 10.63 µm after one month of treatment (*p* = 0.037). No significant changes were observed in mean epithelial thickness (50.80 ± 4.10 vs. 53.00 ± 6.85 µm; *p* = 0.073), minimum epithelial thickness (40.60 ± 6.15 vs. 40.10 ± 4.61 µm; *p* = 0.630), or the superior–inferior epithelial thickness difference (3.00 ± 2.11 vs. 4.40 ± 4.14 µm; *p* = 0.140) (Table 6). The increase in maximum epithelial thickness showed a moderate standardized effect size, while the effect estimates for the other parameters were less precise, with 95% CIs including zero (Table 7). No significant change was observed in the corneal epithelial thickness variability index after treatment (*p* = 0.063) (Table 8).

### 3.4. Comparison of Values After Treatment to the Healthy Controls

Following treatment, patients continued to report more symptoms (*p* < 0.001) and had lower tear production (*p* = 0.010) and tear film stability (*p* = 0.011) compared with healthy controls. Conjunctival hyperemia also remained more pronounced in patients after treatment (*p* < 0.001), whereas no significant differences were observed in blepharitis (*p* = 1.000), MGD (*p* = 0.345), TMH (*p* = 0.860), and corneal fluorescein staining (*p* = 0.502) (Table 9 and Table 10).

Regarding corneal epithelial thickness, no significant differences were observed between patients after treatment and healthy controls in mean (*p* = 0.162), minimum (*p* = 0.791), or maximum epithelial thickness (*p* = 0.171) or in the superior–inferior epithelial thickness difference (*p* = 0.263). In contrast, the corneal epithelial thickness variability index was significantly higher in patients after treatment than in healthy controls (*p* = 0.038) (Table 11 and Table 12).

## 4. Discussion

In this prospective controlled pilot study, we evaluated the efficacy of topical azithromycin as the second-line treatment for patients with mixed DED.

There was a significant improvement in symptoms after one month of treatment, as reported by most participants and also measured by the DEQ-5 questionnaire. Improvement of symptoms is an important clinical outcome of any DED treatment. Of note is also the relatively fast effect already after one month of therapy, and, particularly in chronic DED, the simple treatment protocol used in the study.

There was a significant improvement in two basic tests for DED, the Schirmer test and TBUT, measured non-invasively as NITBUT. These two tests provide evidence of improvement in tear production and tear film stability. In addition, reduced corneal fluorescein staining and increase in maximum corneal epithelial thickness suggest an effect of azithromycin treatment extending beyond the tear film to the ocular surface epithelium.

In line with our expectations, there was also significant improvement in the clinical signs of blepharitis and MGD. These findings are consistent with the expected effects of azithromycin on the eyelids and thus the evaporative component of mixed DED. Conjunctival hyperemia decreased after treatment, but the change did not reach statistical significance, while TMH remained unchanged.

### 4.1. Improvement of Dry Eye Symptoms

Among the primary outcomes, the greatest standardized improvement was observed in patient-reported symptoms, with a large effect size for the reduction in DEQ-5 scores. Although symptoms improved significantly after one month of treatment, the mean DEQ-5 score remained above the threshold of 6 points for symptomatic DED and was significantly higher than that of the healthy control group. These findings indicate an improvement in symptoms, but not their complete resolution. Longer treatment durations, different treatment protocols, or repeated treatment cycles should therefore be investigated in future studies to determine whether further symptomatic improvement can be achieved.

Our findings in patients with mixed DED are comparable to a subgroup of patients with MGD treated with topical azithromycin, analyzed in the systematic review by Tao and Tao, in whom dry eye symptoms improved significantly [38]. Similar improvements after two to four weeks of treatment have also been reported in several studies of patients with blepharitis or MGD [30,31,32,39,40,41,42]. More recently, Shimazaki et al. reported significant improvements in subjective symptoms and several clinical signs of blepharitis and MGD following topical azithromycin treatment; the Dry Eye-related Quality of Life Score (DEQS) also improved significantly by day 28 [33]. Jarvis et al. reported significant improvements in MGD severity and fluorescein tear break-up time following topical azithromycin treatment, with reported improvements in symptoms and lifestyle persisting one year after treatment [34]. In a randomized controlled trial of 36 patients with MGD-associated posterior blepharitis, Arita et al. demonstrated greater improvement in symptoms with topical azithromycin than with preservative-free artificial tears alone [42]. Similarly, Kallab et al. reported a statistically significant improvement in symptoms assessed using the Ocular Surface Disease Index (OSDI) four weeks after initiation of treatment with preservative-free 0.335% hydrocortisone (Softacort, Laboratoires Théa, Clermont-Ferrand, France) in patients with chronic DED [43]. As these studies did not use the DEQ-5 questionnaire, the magnitude of improvement cannot be directly compared with our results. These findings suggest that the one-month treatment regimen was effective in reducing symptoms; however, further studies should evaluate longer treatment protocols, repeated treatment cycles, and combination with other agents for the treatment of mixed DED.

### 4.2. Augmentation of the Tear Film

The mean of Schirmer test I measures increased from 4.27 mm to 9.93 mm after treatment, with a large standardized effect size, indicating significant increase in tear production. More so, the mean NITBUT on Sirius PLUS measures increased from 5.49 s to 10.01 s, with a moderate standardized effect size, which suggests favorable effect of azithromycin on tear film stability in patients with mixed DED.

In the meta-analysis by Tao and Tao, topical azithromycin significantly improved both TBUT and tear production assessed by the Schirmer test in patients with MGD [38]. Similar improvements in both Schirmer test values and tear film stability after one month of treatment have also been reported by Opitz et al. and Balci and Gulkilik in patients with MGD [33,40]. In contrast, Arita et al. reported a significant improvement in NITBUT but no significant change in Schirmer test values after two weeks of treatment [42].

Since exclusively patients with mixed DED were included in our study, the observed improvement in both tests suggests not only a positive effect of azithromycin on MGD and the evaporative DED component, but also an improvement in tear production and the aqueous-deficient component of the disease. Our findings therefore suggest that adjunctive topical azithromycin may also be beneficial in patients with mixed DED, not only in patients with MGD.

TMH reflects the instantaneous tear volume, which is influenced not only by tear secretion but also by tear drainage, blinking, and lower eyelid anatomy [44,45,46]. The constant measures after treatment thus have to be evaluated further in terms of eyelid anatomy and tear drainage. In addition, in the present study, TMH was measured after the Schirmer test, not following the Tear Film and Ocular Surface Society Dry Eye Workshop II (TFOS DEWS II) Diagnostic Methodology Report, which recommends performing ocular surface examinations from the least to the most invasive [44]. Irritation caused by the Schirmer strip may have induced reflex tearing, thereby affecting TMH measurements and potentially masking differences between study visits.

Previous studies have also reported inconsistent associations between TMH and Schirmer test values. Savini et al. demonstrated a significant correlation between the two measurements in a cohort including both patients with DED and healthy controls, whereas Wang et al. did not confirm this association in healthy participants [47,48]. Future studies should therefore perform TMH measurements before the Schirmer test in accordance with TFOS DEWS II recommendations, as well as interpret TMH as a complementary rather than a primary indicator of tear film status.

### 4.3. Ocular Surface Healing and Epithelial Thickness

After the treatment, signs of ocular surface damage decreased significantly. The median Oxford score of cornea fluorescein staining changed from 2 before the treatment to 0 after one month of azithromycin therapy. Although the median conjunctival hyperemia score remained unchanged, the distribution of scores showed a trend toward improvement, but the change did not reach statistical significance (see Section 3.3.3).

Compared with the healthy control group, post-treatment Oxford staining was no longer significantly different; however, conjunctival hyperemia remained more pronounced in patients with mixed DED.

Previous findings regarding corneal fluorescein staining have been less consistent. While the meta-analysis by Tao and Tao and the 30-day study by Opitz et al. demonstrated significant improvement following topical azithromycin treatment, Arita et al. did not observe a significant change after only two weeks of treatment [31,38,41]. These discrepancies may reflect differences in treatment duration, baseline disease severity, or the grading systems used to assess ocular surface staining.

Experimental studies have shown that azithromycin inhibits activation of the NF-κB signaling pathway, which plays a central role in regulating the inflammatory response [24]. These effects may contribute to reduced ocular surface inflammation and successful interruption of the positive feedback loop of the DED and healing of the surface defects.

Corneal epithelial thickness as measured by AS-SS-OCT increased after one month of treatment and thus also proved a positive therapeutic effect of azithromycin on corneal epithelium. However, of the measured parameters the maximum epithelial thickness proved statistical significance, suggests a rather isolated increase in the measured value, which might be explained by the relatively short time of the follow-up [49].

To our knowledge, no previous studies have evaluated the effect of topical azithromycin on corneal epithelial thickness in patients with mixed DED, which precludes direct comparison with our findings. There are heterogeneous results about the baseline epithelial thickness in different stages of DED, but patients with more severe diseases tend to have greater epithelial thickness variability across different studies [14,15,16]. Recent findings also suggest that corneal epithelial thickness profiles may differ according to the underlying DED subtype, with regional differences observed between aqueous-deficient and evaporative DED [50]. Changes in corneal epithelial thickness have also been reported following successful treatment of DED. In a Sjögren syndrome study, increased superior epithelial thickness was measured after 4 weeks of HA artificial tears, whereas treatment with autologous serum reduced epithelial thickness variability [51,52]. However, due to variable reports, measures of and any changes in thickness of corneal epithelium has to be interpretated carefully, keeping in mind that they may also reflect compensatory epithelial hyperplasia or localized epithelial remodeling.

In our study, epithelial thickness variability did not change significantly after treatment but remained significantly greater in patients after treatment than in the healthy control group. This may indicate persistent irregularity of the corneal epithelium despite improvement in other clinical parameters.

An increase in maximum epithelial thickness observed in our study may represent localized epithelial remodeling associated with treatment. However, this interpretation remains speculative. Further longitudinal studies with longer follow-up, repeated epithelial mapping, and point-by-point analysis of epithelial thickness maps are needed to clarify how epithelial thickness changes over time in relation to tear film improvement following topical azithromycin treatment.

### 4.4. Improvement of Eyelids

Following treatment, all assessed clinical signs of eyelid disease improved significantly. The median blepharitis score decreased from 1 to 0, and the median MGD score from 1 to 0.

Following treatment, the median blepharitis and MGD scores reached the lowest possible level, suggesting substantial improvement in these clinical signs. A similar pattern was observed when post-treatment findings were compared with the healthy control group, as differences in blepharitis or MGD evaluation were no longer significant.

Our findings are consistent with the systematic review and meta-analysis by Tao and Tao, which demonstrated significant improvements in eyelid signs, meibomian gland plugging, meibum quality, and corneal fluorescein staining following treatment with topical azithromycin in patients with MGD [38]. Similar improvements in eyelid margin redness, meibomian gland plugging, and meibum quality after two to four weeks of topical azithromycin treatment have also been reported in several clinical studies [30,31,32,39,40,41,42]. In a randomized controlled trial, Luchs et al. demonstrated greater improvement in meibomian gland plugging, meibum quality, and eyelid margin redness with topical azithromycin combined with warm compresses than with warm compresses alone [30].

The observed improvement in clinical signs may be explained by several mechanisms of action of topical azithromycin. The antimicrobial activity of azithromycin may contribute to reduced bacterial colonization of the eyelid margin. Haque et al. demonstrated a significant reduction in eyelid bacterial load after four weeks of topical azithromycin treatment. They also evaluated tear cytokine concentrations, although no significant changes were observed following treatment [32]. Experimental studies have also shown that azithromycin inhibits activation of the nuclear factor kappa B, NF-κB signaling pathway, which plays a central role in regulating the inflammatory response, and promotes differentiation and lipid accumulation in human meibomian gland epithelial cells [24,53]. These effects may contribute to reduced eyelid inflammation, improved meibum quality, and a more stable tear film lipid layer.

Because our study did not assess eyelid bacterial colonization, inflammatory mediators, meibum characteristics, or tear film lipid layer properties, we cannot determine which of these mechanisms contributed most to the observed clinical improvement. Nevertheless, our findings suggest that the previously established efficacy of topical azithromycin in patients with MGD may also extend to patients with mixed DED.

### 4.5. Strengths and Limitations

The strengths of this study include its prospective design with within-subject comparison before and after treatment as well as a control group of healthy individuals, as well as the comprehensive assessment of both subjective and objective outcomes. Treatment response was evaluated using complementary measures of dry eye symptoms, tear production, tear film stability, eyelid disease, ocular surface damage, and corneal epithelial thickness. To our knowledge, this is the first prospective study to evaluate the effects of topical azithromycin on clinical outcomes and corneal epithelial thickness in patients with mixed DED.

In addition to statistical significance, standardized effect sizes and confidence intervals were reported for continuous outcomes, providing a more comprehensive interpretation of treatment effects.

Another strength of the study was the use of non-invasive and standardized imaging techniques. Tear film stability was assessed using automated NITBUT measurements obtained with the Sirius device, thereby avoiding fluorescein instillation and reducing examiner dependency compared with conventional TBUT [44]. The same device was used to capture ocular surface images, allowing standardized grading of clinical signs according to the Efron and Oxford scales. Although these assessments remained subjective, the stored images enabled direct comparison between study visits and retrospective verification of grading. TMH was measured using a digital caliper tool on the stored images, reducing reliance on visual estimation.

The main limitation of this study was the absence of a randomized controlled group and masking of investigators. The non-randomized and unmasked study design, together with the absence of a placebo or active treatment comparator, limits the ability to attribute the observed improvements specifically to topical azithromycin. The relatively small sample size limited the statistical power of the study and the precision of the estimated treatment effects, especially for corneal epithelial thickness measurements were only 10 patients were evaluated. The order of examinations did not strictly follow the TFOS DEWS II Diagnostic Methodology Report and should be adjusted in further studies; TMH assessment should be performed prior to Schirmer testing [44].

The DEQ-5 questionnaire was administered using a translated but non-validated Slovenian version. The same version was used at both baseline and follow-up. The lack of formal linguistic adaptation and psychometric validation of the Slovenian version represents a limitation of the study and may affect the reliability of symptom assessment. A formal process of linguistic adaptation is currently underway to support its future use in clinical practice.

Future research should focus on confirming these findings in larger randomized controlled studies, evaluating the long-term durability of treatment effects, and further investigating the relationship between tear film improvement and corneal epithelial remodeling.

## 5. Conclusions

In this prospective pilot study, one month of treatment with topical azithromycin as a second-line dry eye therapy was associated with significant improvements in symptoms, tear production, tear film stability, eyelid disease, corneal fluorescein staining in patients with mixed DED, and a significant increase in maximum epithelial thickness. To our knowledge, this is the first prospective study to evaluate the effects of topical azithromycin on both clinical outcomes and corneal epithelial thickness in patients with mixed DED. These findings suggest that topical azithromycin may be a useful adjunct to standard therapy in selected patients with mixed DED, particularly in the presence of eyelid disease or MGD, and may also be associated with localized corneal epithelial remodeling. Larger randomized controlled studies with longer follow-up and standardized measurement protocols in accordance with established dry eye disease guidelines are needed to confirm these findings, determine the durability of treatment effects, establish the optimal frequency and duration of treatment, and further investigate changes in corneal epithelial thickness following treatment.

## Figures and Tables

**Table 1:** Baseline characteristics of the study participants.

Characteristics	Mixed DED (*n* = 15)	Healthy Controls (*n* = 10)
Male:female ratio	6:9	4:6
Age (years), mean ± SD (range)	65.7 ± 13.9 (26.1–79.9)	57.7 ± 15.6 (38.8–79.6)

Abbreviations: SD, standard deviation; DED, dry eye disease.

**Table 2:** Changes in the primary outcome measures after one month of topical azithromycin treatment (*n* = 15).

Primary Outcomes	Baseline	Month 1	Mean Change ± SD	95% CI	*p* Value
DEQ-5	13.00 ± 5.07	8.67 ± 5.21	−4.33 ± 3.27	−6.14 to −2.53	<0.001
Schirmer test (mm/5 min)	4.27 ± 2.60	9.93 ± 6.84	5.67 ± 6.14	2.27 to 9.07	0.003
NITBUT (s)	5.49 ± 2.81	10.01 ± 6.48	4.53 ± 7.10	0.59 to 8.46	0.027

Abbreviations: DEQ-5, Dry Eye Questionnaire-5; NITBUT, non-invasive tear break-up time.

**Table 3:** Effect sizes for changes in primary outcome measures.

Outcome	Hedges’ g	95% CI
DEQ-5	−1.254	−1.91 to −0.58
Schirmer test (mm/5 min)	0.873	0.29 to 1.44
NITBUT (s)	0.602	0.067 to 1.12

**Table 4:** Changes in clinical signs after one month of topical azithromycin treatment.

Secondary Outcome	Baseline, Median (IQR)	Month 1, Median (IQR)	*p* Value
Blepharitis (Efron grade)	1 (1–2)	0 (0–0)	<0.001
MGD (Efron grade)	1 (1–2)	0 (0–1)	<0.001
Conjunctival hyperemia (Efron grade)	2 (2–3)	2 (1–2)	0.063
Corneal fluorescein staining (Oxford grade)	2 (1–3)	0 (0–1)	0.002

Abbreviations: IQR, interquartile range; MGD, meibomian gland dysfunction.

**Table 5:** Changes in TMH after one month of topical azithromycin treatment.

Secondary Outcome	Baseline	Month 1	Mean Change ± SD	95% CI	*p* Value
TMH (mm)	0.36 ± 0.18	0.36 ± 0.17	0.0027 ± 0.20	−0.11–0.11	0.960

Abbreviations: TMH, tear meniscus height.

**Table 6:** Changes in corneal epithelial thickness after one month of topical azithromycin treatment (*n* = 10).

Outcome	Baseline	Month 1	Mean Change ± SD	95% CI	*p* Value
Mean epithelial thickness (µm)	50.80 ± 4.10	53.00 ± 6.85	2.20 ± 3.43	−0.25 to 4.65	0.073
Maximum epithelial thickness (µm)	59.10 ± 5.67	64.30 ± 10.63	5.20 ± 6.73	0.39 to 10.01	0.037
Minimum epithelial thickness (µm)	40.60 ± 6.15	40.10 ± 4.61	−0.50 ± 3.17	−2.77 to 1.77	0.630
Superior–inferior epithelial thickness difference (µm)	3.00 ± 2.11	4.40 ± 4.14	1.40 ± 2.76	−0.57 to 3.37	0.140

**Table 7:** Effect sizes for changes in corneal epithelial thickness parameters (*n* = 10).

Outcome	Hedges’ g	95% CI
Mean epithelial thickness (µm)	0.587	−0.052 to 1.20
Maximum epithelial thickness (µm)	0.706	0.040 to 1.34
Minimum epithelial thickness (µm)	−0.144	−0.71 to 0.43
Superior–inferior epithelial thickness difference (µm)	0.464	−0.152 to 1.06

**Table 8:** Change in the corneal epithelial thickness variability index after treatment (*n* = 10).

Outcome	Baseline, Median (IQR)	Month 1, Median (IQR)	*p* Value
Corneal epithelial thickness variability index (µm)	3.00 (2.00–3.25)	3.00 (2.75–5.00)	0.063

**Table 9:** Comparison of DEQ-5 scores between patients after treatment and healthy control groups.

Outcome	Month 1 Treatment Group (*n* = 15)	Healthy Control Group (*n* = 10)	Mean Difference	95% CI	*p* Value
DEQ-5	8.67 ± 5.21	2.00 ± 1.63	6.67	3.64 to 9.69	<0.001

**Table 10:** Comparison of clinical outcomes between the treatment and healthy control groups.

Outcome	Month 1 Treatment Group (*n* = 15), Median (IQR)	Healthy Control Group (*n* = 10), Median (IQR)	*p* Value
Schirmer test (mm/5 min)	10 (4–12)	20 (10–35)	0.010
NITBUT (s)	8.5 (3.7–17.9)	18.0 (12.9–18.1)	0.011
Blepharitis (Efron grade)	0 (0–0)	0 (0–0)	1.000
MGD (Efron grade)	0 (0–1)	0 (0–0)	0.345
Conjunctival hyperemia (Efron grade)	2 (1–2)	0.5 (0–1)	<0.001
TMH, mm	0.32 (0.28–0.48)	0.36 (0.24–0.38)	0.860
Corneal fluorescein staining (Oxford grade)	0 (0–1)	0 (0–1)	0.502

**Table 11:** Comparison of mean and minimum corneal epithelial thickness between patients after treatment and the healthy control group.

Outcome	Month 1 Treatment Group (*n* = 15)	Healthy Control Group (*n* = 10)	Mean Difference	95% CI	*p* Value
Mean epithelial thickness (µm)	52.40 ± 8.77	48.90 ± 2.42	3.50	−1.54 to 8.54	0.162
Minimum epithelial thickness (µm)	39.47 ± 4.60	39.90 ± 2.69	−0.43	−3.78 to 2.91	0.791

**Table 12:** Comparison of maximum corneal epithelial thickness, the superior–inferior difference, and the corneal epithelial thickness variability index between patients after treatment and the healthy control group.

Outcome	Month 1 Treatment Group (*n* = 15), Median (IQR)	Healthy Control Group (*n* = 10), Median (IQR)	*p* Value
Maximum epithelial thickness (µm)	63 (55–69)	57 (54–59.75)	0.171
Superior–inferior epithelial thickness difference (µm)	4 (2–5)	2.5 (1–4.25)	0.263
Corneal epithelial thickness variability index (µm)	3 (3–5)	3 (2–3)	0.038

## Data Availability

De-identified clinical, laboratory, and imaging data supporting the findings of this study are available from the corresponding authors upon reasonable request, subject to institutional data-sharing policies.

## Abbreviations

The following abbreviations are used in this manuscript:

DED	Dry Eye Disease
DEQ-5	Dry Eye Questionnaire-5
IL	Interleukin
MGD	Meibomian gland dysfunction
NF-κB	nuclear factor kappa B
NITBUT	non-invasive tear break-up time
SS OCT	swept-source optical coherence tomography
TFOS DEWS II	Tear Film and Ocular Surface Society Dry Eye Workshop II
TMH	tear meniscus height
TNF-α	tumor necrosis factor-alpha
